# Pre-Ribosomal RNA Processing in Human Cells: From Mechanisms to Congenital Diseases

**DOI:** 10.3390/biom8040123

**Published:** 2018-10-24

**Authors:** Maxime Aubert, Marie-Françoise O’Donohue, Simon Lebaron, Pierre-Emmanuel Gleizes

**Affiliations:** Laboratoire de Biologie Moléculaire Eucaryote, Centre de Biologie Intégrative (CBI), Université de Toulouse, CNRS, UPS, 31000 Toulouse, France; aubert@ibcg.biotoul.fr (M.A.); odonohue@ibcg.biotoul.fr (M.-F.O.); lebaron@ibcg.biotoul.fr (S.L.)

**Keywords:** ribosomal RNAs (rRNAs), endonucleases, exonucleases, RNA processing, ribosomopathies, Diamond–Blackfan anemia, ribosomal stress

## Abstract

Ribosomal RNAs, the most abundant cellular RNA species, have evolved as the structural scaffold and the catalytic center of protein synthesis in every living organism. In eukaryotes, they are produced from a long primary transcript through an intricate sequence of processing steps that include RNA cleavage and folding and nucleotide modification. The mechanisms underlying this process in human cells have long been investigated, but technological advances have accelerated their study in the past decade. In addition, the association of congenital diseases to defects in ribosome synthesis has highlighted the central place of ribosomal RNA maturation in cell physiology regulation and broadened the interest in these mechanisms. Here, we give an overview of the current knowledge of pre-ribosomal RNA processing in human cells in light of recent progress and discuss how dysfunction of this pathway may contribute to the physiopathology of congenital diseases.

## 1. A Renewed Perspective on Pre-rRNA Processing in Human Cells

**Figure 1:**
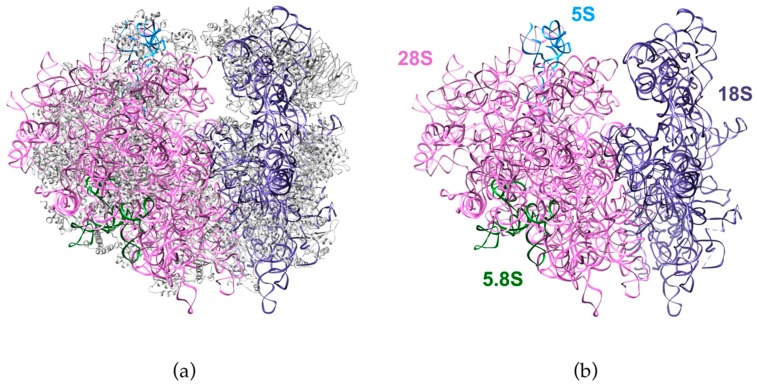
Structure of the human ribosome solved by cryo-electron microscopy. The figure shows the ribosomal RNAs in the large subunit (28S: pink, 5.8S: green, 5S: blue) and in the small subunit (18S: purple). (**a**) Structure with the ribosomal proteins (grey). (**b**) Ribosomal RNAs only. Adapted from Protein Data Bank (PDB) file 4UG0 [19].

Initially described by Georges Palade in 1955 from electron microscopy micrographs [1], ribosomes have been the focus of continuous research for the past 60 years. In addition to their key role in protein synthesis, the complex mechanisms of the synthesis of these prototypic ribonucleoprotein particles have been under constant investigation. The picture of the ribosome structure gradually emerged from pioneering biochemical and structural works, until its observation by X-ray crystallography at the turn of the century delivered an atomic view of the intricate relationships between the RNAs and the proteins composing the ribosome, first in prokaryotes [2,3,4] and later in eukaryotes [5,6,7]. The advent of cryo-electron microscopy (cryo-EM) as a high-resolution technique for structural biology recently bypassed the need for crystals and delivered stunning images of ribosomes and their maturation precursors, both from unicellular organisms [8,9,10,11,12,13,14,15,16,17] and human cells [18,19,20] (Figure 1).

**Figure 2:**
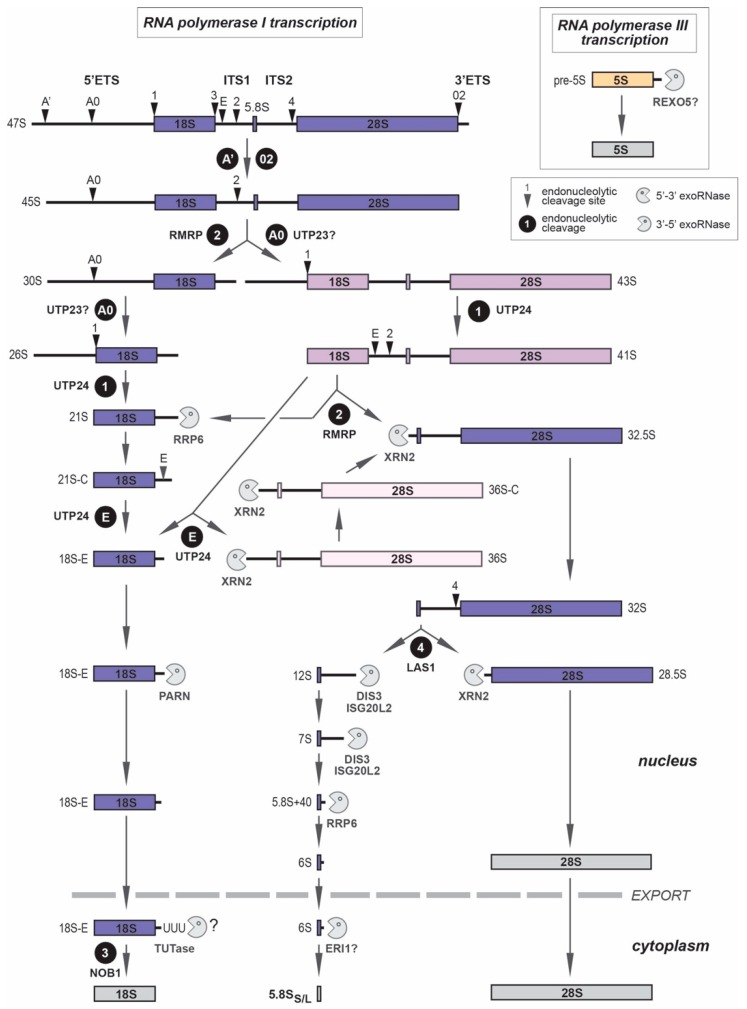
Pre-ribosomal RNA processing in human cells. Three of the four ribosomal RNAs arise from a long primary transcript (47S pre-rRNA) synthesized by RNA polymerase I from loci containing highly-repeated ribosomal DNA (rDNA) genes (also called nucleolar organizer regions or NORs) located in the cell nucleoli. The sequences of 18S (1870 nucleotides (nt)), 5.8S_S/L_ (157 and 162 nt, respectively), and 28S rRNAs (>5000 nt) are flanked by external (5′-ETS, 3656 nt; 3′ ETS, 345 nt) and internal transcribed spacers (ITS1, 1090 nt; ITS2, 1155 nt), which are gradually removed by endo- and exonucleases. Depending on the relative kinetics of endoribonucleolytic cleavage, various rRNA precursors are formed. In the main maturation pathway (pre-rRNAs colored in violet), cleavage at site 2 occurs prior to cleavage at site A0. Less abundant precursors are characteristic of alternative (pale lavender) or minor (pink) routes. The 5S rRNA (121 nt) is transcribed by RNA polymerase III from repeated gene copies located on chromosome 1. As little is known about the maturation of 5S precursors in humans (depicted in orange) [34], data recently obtained in *Drosophila* were used [35]. When identified, endoribonucleases are quoted in black, and 5′-3′ or 3′-5′ exoribonucleases in grey. Question marks refer to uncertain enzymatic activities (UTP23), or enzymes found in other organisms which could putatively play similar functions in humans (REXO5, ERI1). Site E is also called 2a by some authors.

Confirming decades of biochemical studies, these images highlight the central role of ribosomal RNAs (rRNAs) as the scaffold of the ribosomal subunits, but also as the hub of the catalytic activities of ribosomes in protein synthesis. Eukaryotic ribosomes are made of four RNAs that share a large degree of conservation, but have acquired additional domains, called extension segments, during evolution. In human ribosomes, the 18S rRNA assembles with 33 ribosomal proteins (RPSs) to form the 40S ribosomal subunit or small subunit (SSU), while the 5S, 5.8S, and 28S rRNAs associate with 47 ribosomal proteins (RPLs) to assemble the 60S or large subunit (LSU). The 18S, 5.8S, and 28S pre-rRNAs are formed from a common polycistronic transcript following several RNA processing steps, which generate multiple intermediate RNAs called pre-rRNAs (Figure 2). Pre-rRNAs were initially described in mammalian cells, but the intricate molecular mechanisms underlying their maturation were largely deciphered in yeast *Saccharomyces cerevisiae*, which became the gold standard for these studies [21,22]. The wealth of results obtained in yeast on this conserved process, together with the development of new molecular genetic tools, high-content screening methods, and proteomics analyses, has recently allowed significant advances on the study of pre-rRNA processing in human cells and revealed a higher complexity of these mechanisms when compared to yeast [23,24,25,26,27]. This renewed interest in human pre-rRNA maturation has also been fueled by the discovery of a growing class of inheritable diseases, called ribosomopathies, that are characterized by defects in ribosome production or function [28,29]. Recent reviews have described in great details our current knowledge of pre-rRNA processing in various organisms, including yeast, mammalian cells, and plants [30,31,32]. Here, our aim is to propose an overview of pre-rRNA processing in human cells, and discuss how dysfunctions of this process are linked to human diseases.

## 2. Human Pre-rRNA Processing Is Both Hierarchical and Modular

The human primary pre-rRNA, or 47S pre-rRNA, is synthesized by RNA polymerase I from the ~400 head-to-tail tandem repeats of ribosomal DNA (per diploid genome) located on the short arm of the five acrocentric chromosomes 13, 14, 15, 21, and 22. Synthesis of pre-rRNAs triggers self-assembly of the nucleolus around these genomic loci, also called nucleolar organizer regions (NORs), through the recruitment of a large array of proteins and noncoding RNAs taking part in pre-rRNA processing. Within this primary pre-rRNA transcript, the 18S, 5.8S, and 28S rRNAs are flanked by the 5′ and 3′ external transcribed spacers (ETS) and two internal transcribed spacers (ITS1 and ITS2; Figure 2). The transcribed spacers contain several cleavage sites targeted by endonucleases that act sequentially to free the rRNAs. While the rRNA sequences are conserved among eukaryotes, the sequence and the length of the transcribed spacers strongly diverge. Despite these differences, bioinformatic analyses indicate that folding of the transcribed spacers positions the endonucleolytic cleavage sites in similar secondary structure elements, which favors their processing. For example, the A0 and 1 cleavage sites in 5′-ETS are predicted to frame the base of a large stem in humans [33], as observed in yeast [12,14,15], although the sequence separating these two sites is over 20 times longer in humans (2010 nucleotides (nt)) than in yeast *S. cerevisiae* (92 nt).

Figure 2 depicts the gradual elimination of the transcribed spacers in the human primary ribosomal transcript by the sequential action of endo- and exoribonucleases. Several nucleases involved in pre-rRNA processing were initially discovered in yeast. Human orthologs of these enzymes are also involved in rRNA maturation and the overall processing scheme has been conserved through evolution [31,32]. However, pre-rRNA processing in mammalian cells turns out to be more complex than in yeast, as it requires exonucleolytic steps following almost all endonucleolytic cleavages [36,37,38,39]. The intricacy of the maturation scheme is also increased by the modularity of the processing events: while some processing steps obey a hierarchical order, others appear to be independent from one another. For example, after initial cleavage of the 47S pre-rRNA at sites A’ in the 5′-ETS and 02 in the 3′-ETS, the 45S pre-rRNA is processed either by further elimination of the 5′-ETS, or by cleavage of the ITS1 at site 2 (Figure 2). Most defects in 5′-ETS processing neither block site 2 cleavage nor affect subsequent maturation of the 5.8S and 28S rRNAs [40]. Conversely, partial or full removal of the 5′-ETS may occur before ITS1 cleavage, which produces the characteristic 43S or 41S pre-rRNAs. But while partly flexible, the cleavage order also includes clear hierarchical links. For example, endonucleolytic processing at site E (also called 2a) in the ITS1 only occurs after full removal of the 5′-ETS by cleavage at sites A0 and 1. Hence, cleavage of the ITS1 may also directly take place at site E, albeit infrequently, which produces the 36S precursor [37,38], but this requires prior removal of the 5′-ETS. Similarly, endonucleolytic cleavage of the ITS2 requires prior cleavage of the ITS1. Because of these coexisting pathways, the ratio between the rRNA precursors may vary among cell types and are drastically modified in some pathological contexts [41,42] or during viral infection [43]. These modified pre-rRNA patterns indicate changes in the relative kinetics of the processing steps and may reflect defects in ribosome biogenesis. Further work is needed to demonstrate whether changing the order of cleavages may impact ribosome maturation per se and lead to structural variability in ribosomes, for example by modifying the kinetics, and thereby the pattern, of rRNA post-transcriptional modifications [44].

## 3. Pre-rRNA Processing Is Coordinated with RNA Folding and Modification

Cleavage of pre-rRNAs is paralleled by chemical modification of around 200 nucleotides within the emerging rRNA sequences. Most of these modifications are pseudouridylations and 2′-*O*-ribose methylations that are guided and catalyzed by two families of small nucleolar RNPs, respectively called H/ACA box and C/D box snoRNPs [45]. Each modification is performed by a particular snoRNP that combines a set of core proteins with a specific small guide RNA (snoRNA) hybridizing around the position to modify. Each complex includes either the pseudouridyl synthase dyskerin (H/ACA box snoRNPs), or the methyltransferase fibrillarin (C/D box snoRNPs). In addition, a few modifications are catalyzed by specific enzymes [44,46]. As a general rule, modifications take place early in the maturation process. They target nucleotides located in functionally important regions of the ribosome, including the peptidyl transferase center or the decoding center [47]. Human rRNAs contain an additional layer of nucleotide modifications when compared to yeast [47]. New modifications were even proposed based on the recent determination of the human ribosome structure at high-resolution by cryo-EM [48], but they were not confirmed by RNA mass spectrometry [47]. The function of these modifications is not fully understood, but they appear to take part in the structure and the reactivity of the rRNAs [44,46]. SnoRNAs that guide modifications were not found to be required for cell viability; however, knockdown of some of them prevents normal development of zebrafish embryos [49].

**Figure 3:**
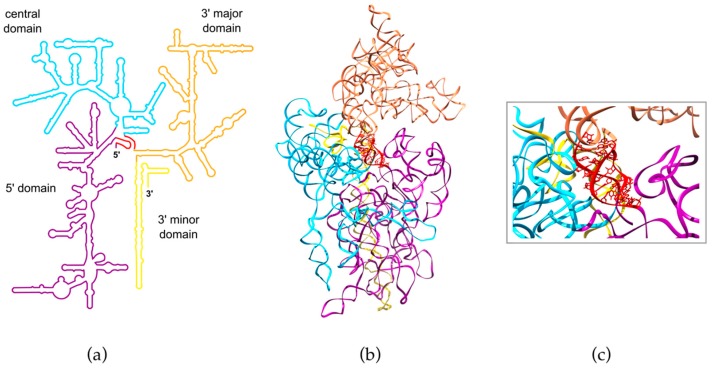
Folding of the human 18S rRNA. The 18S rRNA folds into 45 helices that distribute into four structural domains distinguished here by different colors. These domains assemble around the pseudoknot (red) formed by nucleotides located at the 5′ end that hybridize with nucleotides located ~1200 residues farther down in the primary sequence, at the junction between the central domain and the 3′ major domain. (**a**) Secondary structure of the 18S rRNA. (**b**) Structure of the 18S rRNA within the 40S subunit. (**c**) Enlarged view of the pseudoknot. The 3D structure in (**b**,**c**) was generated from PDB file 4UG0 [19].

Removal of the transcribed spacers and nucleotide modifications take place concomitantly with the folding of pre-rRNAs and their assembly with ribosomal proteins (RPs). Most of the nucleotides in rRNAs are base-paired within the numerous helices that shape the subunits (45 helices in the human 18S rRNA and over 100 in the 60S subunit rRNAs, Figure 3). These helices form several structural subdomains that interlock in a highly controlled manner during maturation of the subunits. Hence, the tertiary structure of the 18S rRNA requires formation of a “pseudoknot”, in which distant sites in the 18S rRNA sequences hybridize with each other (Figure 3). As the nexus of 18S rRNA architecture, folding of this pseudoknot is tightly coordinated with pre-rRNA processing. Similarly, it was shown in yeast that folding of the 25S rRNA (equivalent to the human 28S rRNA) operates gradually through the constitution of subdomains that assemble together like petals around a central node. Timing of folding must be tightly controlled to allow the hierarchical association of these subdomains, which starts with long-range interactions between 5′ and 3′ regions of the 25S rRNA [50,51]. Pre-rRNA folding in turn can directly control the activity of nucleases. Hence, the endonuclease NOB1, which cleaves at the 18S rRNA 3′ end (site 3), is found in pre-40S particles but is maintained out of reach of its cleavage site, as shown in yeast [16,52,53] or humans [18,20]. Structural remodeling of the pre-40S particle at the very end of the maturation process brings NOB1 close enough to its substrate, thus triggering the final step of 18S rRNA formation [20].

## 4. The Role of Ribosomal Proteins and Ribosomal Assembly Factors

Folding and processing of the nascent pre-rRNAs is controlled by both association with RPs and sequential interaction with a large number of ribosomal assembly factors (RAFs). As primary constituents of the ribosome, most RPs are necessary for the synthesis of the subunits and their incorporation is intimately linked to pre-rRNA folding and recruitment of the RAFs [21,22]. Hence, depletion of 31 out of 33 human RPs of the small subunit with small interfering RNAs (siRNAs) blocks pre-rRNA maturation of the 18S rRNA [18,40]. RAFs temporarily associate with the forming subunits and play multiple enzymatic, structural, and regulatory functions. They include nucleases catalyzing the removal of the transcribed spacers, helicases and chaperones participating in the folding of RNA molecules, modifying enzymes (methyl- or acetyltransferases) catalyzing a subset of nucleotide modifications, ATPases providing mechanical energy for structural remodeling of the particles, GTPases acting as molecular switches, as well as nuclear export factors to transport pre-ribosomal particles to the cytoplasm at the end of the maturation process. Structures of pre-ribosomal intermediates in bacteria and eukaryotes have also shown that RAFs pace the maturation steps, for example by isolating pre-rRNA domains that must fold independently, by assessing the correct folding of the structure, or by acting as place holders to prevent premature interactions with late-associating RPs or components of the translation machinery [8,54]. Several RAF modules were identified from the determination of the composition of ribosomal particles at various maturation stages. For example, a large subset of factors associates co-transcriptionally with the emerging 5′ part of the pre-rRNA (5′-ETS and 18S domains) and forms a large RNP particle, called the SSU processome, which is mostly required for elimination of the 5′-ETS and maturation of the 18S rRNA [55,56,57,58]. The SSU processome includes the U3 C/D box snoRNP, whose RNA base-pairs both with the 5′-ETS and the 18S rRNA. By bridging these distant domains, U3 is instrumental in coordinating formation of the 18S rRNA pseudoknot with the early cleavages in the 5′-ETS and ITS1 (A0, 1, and E). This crucial role makes U3 essential for 18S rRNA production and cell viability, unlike most snoRNAs. Similar to U3, a few other snoRNPs (U8, U14, U17, and U22) do not catalyze nucleotide modification, but play a crucial role at some pre-rRNA processing steps by chaperoning the pre-rRNAs [45]. For example, the U17/snR30 snoRNA contains two evolutionarily conserved sequence elements that hybridize within the 18S rRNA and are critical for removal of the 5′-ETS, as shown in yeast [59]. U17 may serve as a platform to recruit several RAFs, including the putative endonuclease UTP23 [60].

## 5. A Tour of the 18S rRNA Maturation Scheme

In the main pre-rRNA processing pathway observed in mammalian cells, cleavage of the 45S pre-rRNA at site 2 separates the 30S pre-rRNA, precursor to the 18S rRNA, from the large subunit RNAs (Figure 2). This cleavage is performed by RNase MRP (mitochondrial RNA processing) or RMRP [61]. Due to its similarity with RNase P, RNase MRP likely acts as a ribozyme. Site 2 cleavage also requires several RAFs, including a set of them that are involved in formation of the 60S subunit and associate with the ITS1 [38,62,63]. Formation of the 18S rRNA then requires elimination of the 5′-ETS and the ITS1 at the 3′ end. The 5′-ETS contains three cleavage sites: A’ (also called 01) and A0 located within the 5′-ETS, and site 1, which defines the 5′ end of the 18S rRNA. As indicated above, cleavage at A’ takes place very early, before processing at site 2 and independently from cleavage at A0 and 1 [40,58]. The enzyme that cleaves A’ is unknown, but is likely to be found among the components of the SSU processome. This cleavage site, only found in metazoans so far, is located upstream of a conserved binding site for snoRNA U3 [64], but its functional role remains unclear. Unlike A’, cleavages at sites A0 and 1 are coordinated with one another, as indicated by the low abundance of 43S and 26S pre-rRNAs relative to other precursors (Figure 2), two species in which the 5′-ETS is cleaved at site A0 but not at site 1. Although separated by approximately 2000 nucleotides, sites A0 and 1 are predicted to be in close spatial proximity after formation of a very large stem in the 5′-ETS [33]. In addition, cleavage of the ITS1 at site E is subordinated to cleavage at site 1. A component of the SSU processome, hUTP24, was recently identified as the endonuclease for sites 1 and E [62,65,66]. hUTP24 contains a PIN (PilT N-terminus) domain, which is present in several endonucleases. Presence of hUTP24 is essential for processing at sites A0 and 1, as well as site E in the ITS1, but its catalytic activity is only required for processing at sites 1 and E, and not A0 [65,66]. The endonucleolytic activity of hUTP24 was evidenced in vitro on an RNA template containing yeast site A2, the equivalent to site E [65]. Interestingly, the 5′ end of the 18S rRNA was still residually processed after hUTP24 knockdown, but started 2 nucleotides downstream of the normal extremity, which may indicate the involvement of a 5′–3′ exonuclease [65,66]. The endoribonuclease that cleaves site A0 has not been identified yet. Mutation of protein hUTP23, which bears an incomplete PIN domain, leads to accumulation of 30S pre-rRNA, indicating that processing at site A0 is impaired [60]. It remains to show whether hUTP23 is a bona fide endonuclease or a cofactor required for cleavage.

Removal of the 5′-ETS and ITS1 cleavage at site 2 yields the 21S pre-rRNA. This precursor is processed at its 3′ end through the sequential action of endo- and exonucleases [23,36,37,38,39,67]. First, the 3′ end of the ITS1 is trimmed by the exosome, and more specifically by RRP6 [23,37,38], which yields the 21S-C. Progression of the exonuclease appears to be stopped by a highly conserved domain in mammalian ITS1 sequences that may adopt a particular fold and/or bind RAFs, thus forming a roadblock [37]. It was also proposed that exonucleolytic trimming could continue past this point. Next, endonucleolytic cleavage of the 21S-C pre-rRNA at site E generates the 18S-E precursor. This step, likely catalyzed by hUTP24 (as is site 1), leaves around 80 nucleotides of ITS1 [37], which are gradually shortened by a 3′–5′ exonuclease. Recent reports have identified Poly(A)-specific ribonuclease (PARN) [39,67] as the enzyme catalyzing this step. PARN was initially characterized as a deadenylase involved in mRNA turnover, but this conclusion has been recently questioned [68,69]. More recent works have shown the implication of PARN in the maturation of several noncoding RNAs, including snoRNAs [70], small Cajal body-specific RNAs (scaRNAs) [68], micro RNAs (miRNAs) [71], Piwi-interacting RNAs (piRNAs) [72,73], and the telomerase RNA component [74,75,76]. Despite the high G/C content of the ITS1, PARN can trim the ITS1 in vitro and its conspicuous nucleolar localization is fully consistent with a major role in ribosome biogenesis [39]. PARN action appears to be primed by oligoadenylation of the 18S-E 3′ end in the nucleolus by PAPD5 [39]. The pre-40S particles then leave the nucleus and are exported to the cytoplasm [77]. Nuclear export does not strictly depend on 18S-E trimming by PARN, but it is delayed upon PARN depletion [39]. In the cytoplasm, the 18S-E pre-rRNA is cleaved by the endonuclease NOB1 to generate the 18S mature rRNA [37,38,78]. However, this endonucleolytic cleavage is preceded by further trimming of the 18S-E 3′ end by a yet-to-be identified exonuclease [37]. Interestingly, the 3′ end of cytoplasmic 18S-E pre-rRNA is oligouridylated [37,39]. The exact function of oligouridylation in 18S rRNA maturation remains to be established, but it could prime the action of the exonuclease processing the ITS1, as shown for the turnover of mRNAs and miRNA let-7 by DIS3L2 [79].

## 6. Processing of the Large Subunit rRNAs

In addition to the 18S rRNA precursors, ITS1 cleavage at site 2 generates the 32.5S pre-rRNA, which contains the 5.8S and 28S rRNAs. The ITS1 is rapidly removed by the 5′–3′ exoribonuclease XRN2, which forms the 5′ ends of the long and short forms of the 5.8S rRNA [37,38,80]. Cleavage of the ITS2 at site 4 in the 32S pre-rRNA by endonuclease Las1 then gives rise of the 12S and the 28.5S pre-rRNA [81], the precursors to the 5.8S and the 28S rRNAs, respectively. Cleavage at site 4 is then followed by exonucleolytic processing of the resulting precursors. The ITS2 domain forming the 5′ end of the 28.5S pre-rRNA is trimmed by XRN2 to form the 28S rRNA [80]. The 12S pre-rRNA in turn is sequentially digested by several 3′–5′ exonucleases to form the mature 3′ end of the 5.8S rRNA. Depletion of exosome subunits, including its catalytic subunit DIS3, or of the exosome cofactors leads to accumulation of intermediate processing fragments between the 12S and 7S pre-rRNAs [82]. The exonuclease ISG20L2 was also proposed to take part in the 3′–5′ trimming of the 12S pre-rRNA [83]. Another mechanism was also proposed to generate the 7S precursor through endonucleolytic cleavage by a yet unknown enzyme [84,85]. Then, conversion of the 7S precursor to 6S depends on the nuclear exosome [23]. The last nucleotides in 3′ of the 5.8S rRNA precursor are likely to be removed by exonuclease ERI1, as shown in mouse [86]. In yeast and in *Xenopus laevis*, this 5.8S rRNA final maturation step takes place in the cytoplasm, which still needs to be demonstrated in mammalian cells.

## 7. 5S rRNA, the Fourth Musketeer

Unlike the 47S pre-rRNA, the precursor to the 5S rRNA is transcribed by RNA polymerase III. In humans, the *RNA5S* genes encoding 5S rRNA are tandemly repeated on chromosome 1. While this chromosome is distinct from ribosomal DNA, the *RNA5S* genes are localized in close proximity to nucleoli [87]. Synthesis of the 5S rRNA requires a specific regulatory factor called transcription factor IIIA (TFIIIA). TFIIIA associates with the general class III initiation factors TFIIIB and TFIIIC on the 5S gene promoter and stimulates transcription [88,89]. Notably, the basal promoter element necessary for 5S rRNA gene transcription is located in the transcribed region. Transcription starts directly at the 5′ end of the 5S rRNA, but the primary transcript bears a uridine-rich 3′ extension [90,91]. This 5S precursor, called 5S*, is recognized by the La protein, which associates with diverse RNA polymerase III transcripts [92]. La has affinity for uridylates in 3′ and acts as a chaperone. The 5S* RNA 3′ end is processed by a 3′–5′ exonuclease, which was recently identified as REXO5 in *Drosophila* [35]. This exonuclease is evolutionary conserved, and its human ortholog is located in the nucleolus [35,93], which supports a similar role in human 5S* RNA processing. However, in mouse, Rexo5 is not essential to viability and fertility, and it does not concentrate in the nucleolus [93]. After processing of the 3′ end, the 5S rRNA is associated with the ribosomal protein L5 (RPL5) in a complex that does not include La [94]. In mammalian cells, formation of this 5S-RPL5 complex was proposed to take place in the cytoplasm, but this point remains to be fully clarified [94]. This complex is then addressed to the nucleolus where it is incorporated into the 60S particles. In yeast, incorporation of the 5S rRNA into the nascent ribosome requires that the 5S RNP includes both Rpl5 and Rpl11 [95]. A specialized importin called Syo1 mediates nuclear import of Rpl5 and Rpl11 and likely chaperones the assembly with the 5S RNA [96]. Incorporation of the 5S RNP into pre-60S particles depends on association with two RAFs, Rpf2, and Rrs1 [97,98]. After binding to the pre-60S particle, the 5S RNP operates a remarkable 180° rotation to reach its final position [10]. The human orthologs of Rrs1 (RRS1) or Rpf2 (BXCD1) do not appear to be strictly required for the recruitment of the 5S into pre-60S particles in human cells [99], but knockdown of these proteins strongly alters nucleolar integrity [100], suggesting that they also play a role in ribosome biogenesis, putatively in 5S RNP final positioning within the subunit. In contrast, the human 5S RNP must include RPL11 to be incorporated into the 60S precursors [99]. Importantly, mammalian cells contain a large pool of free 5S-RPL5 RNPs, representing ~30–50% of total 5S rRNA [94,99,101]. Large amounts of ribosome-free 5S RNA in 7S and 42S RNPs were also evidenced in the cytoplasm of amphibian oocytes and likely correspond to storage (discussed in a previous paper [94]). In mammalian cells, the free 5S-RPL5-RPL11 RNP has ribosome-independent functions in cell cycle regulation, as shown by its capacity to modulate the action of p53 [99,102] (discussed below). This potentially important function, together with the essential role of the 5S RNP in ribosome formation, contrast with the relative lack of knowledge on the maturation, processing, and intracellular dynamics of the 5S rRNA in human cells.

## 8. Defects of Pre-rRNA Processing in Congenital Diseases: The Case of Diamond–Blackfan Anemia

**Figure 4:**
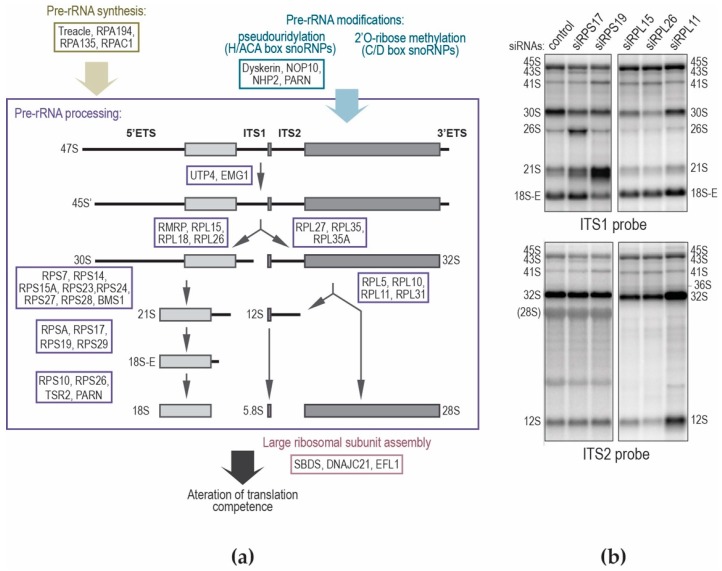
Impairment of ribosome production in human diseases. (**a**) Ribosome production is impaired in a growing number of human diseases through global reduction of rRNA synthesis by RNA polymerase I (yellow ochre), defective pre-rRNA processing steps (purple), decrease of nucleotide chemical modifications (blue), or assembly of 60S ribosomal subunits (pink). The pre-rRNA maturation step that is affected is indicated (see also Table 1). (**b**) Examples of modification of the pre-rRNA pattern in HeLa cells after knockdown of various RPs with siRNAs. Total RNAs were analyzed by northern blot as previously described [40]. Pre-rRNA precursors to the small and large ribosomal subunits were revealed with probes complementary to ITS1 or ITS2.

Ribosomopathies constitute a growing class of diseases characterized by impaired ribosome production and/or impaired ribosome function (Table 1). Such disorders are likely to affect the quantity of ribosomes, when dysfunction of specific pre-rRNA maturation steps alters the production of one of the ribosomal subunits, or their quality by changing protein composition or rRNA modification pattern in at least a subset of ribosomal subunits. Although ribosome biogenesis is ubiquitous, these disorders are characterized by tissue and lineage-specific manifestations, including several inherited bone marrow failures. Patients can suffer from various developmental abnormalities and may present an increased risk of cancer. Diamond–Blackfan anemia (DBA; OMIM #105650) is the archetype of congenital ribosomopathies, not only for historical reasons, but also because its link to defects in ribosome biogenesis was clearly established. This rare disorder (5–7 new cases per million live births) is characterized by pure red cell aplasia [28,103] and patients usually present with severe anemia in the first year of life. Approximately 40 to 50% of affected individuals have growth retardation and may bear congenital abnormalities, such as craniofacial, upper limb, heart, or genitourinary defects. This disease, inherited in an autosomal dominant pattern, is primarily caused by haploinsufficiency of several RP-encoding genes [104,105]. The spectrum of DBA mutations includes missense, nonsense, frameshift, or splice site mutations (accounting for <60% of patients), as well as larger genomic deletions affecting RP loci (10–20% of patients). Most of these mutations prevent mRNA synthesis or stability, or result in protein degradation, thus leading to a net deficit in the production of the corresponding RP. It was shown that missense mutations in *RPS19* that do not destabilize the protein affect domains interacting with the rRNA and hamper incorporation of the mutated protein in nascent ribosomal subunits, thereby again leading to deficit in functional RP for ribosome biogenesis [106,107]. So far, we do not know of any case of a mutated RP incorporated in ribosomal subunits in DBA. Most RPs are essential for ribosome biogenesis in yeast [50,108] as in human cells [26,40,100]. Their deficit impedes specific pre-rRNA processing steps and provokes accumulation of pre-ribosomal particles blocked at a particular maturation step (Figure 4). Accordingly, the impact of DBA mutations in RP genes on ribosome biogenesis can be demonstrated by assessing changes in pre-rRNA processing. Analysis by northern blot of total RNAs from DBA patient cells reveals unbalanced pre-rRNA profiles (relative to unaffected individuals), providing molecular signatures specific to each haploinsufficient RP [41,42,109,110]. This analysis is central when characterizing new DBA-linked mutations, and can contribute to the molecular diagnosis of the disease [111,112,113]. Deficit of the ribosomal subunit corresponding to the mutated RP gene can be confirmed by centrifugation analysis of ribosomes on sucrose gradient [112].

To date, nineteen RP genes have been linked to DBA (Table 1), four of which (*RPS7*, *RPS28*, *RPS27*, and *RPL27*) still lack formal functional validation that ribosome biogenesis is impaired in these patients. It should be mentioned that three non-RP genes have been identified in a few rare DBA pedigrees. First, a missense mutation affects the X-linked *TSR2* gene [114]. The homonymous RAF encoded by this gene is required for incorporation of RPS26 into 40S subunits, which easily explains why loss-of-function of *TSR2* phenocopies haploinsufficiency of *RPS26*, one of the most frequent RP genes affected in DBA. The second one is *GATA1*, which encodes a transcription factor essential for erythrocyte differentiation [115,116,117]. DBA mutations in *GATA1* do not affect pre-rRNA processing (our unpublished results), but affect GATA1 translation by affecting its initiation codon. While the classification of these patients as DBA cases is debated, their clinical picture is close enough to suggest that defects in GATA1 translation in this disease may explain the erythroid lineage-specific phenotype [118,119]. Finally, a recent study has reported nine individuals diagnosed with DBA with biallelic mutations in *CECR1*, encoding the adenosine deaminase ADA2 [105]. The involvement of ADA2 in nucleotide metabolism may provide a link with rRNA synthesis, which is the most demanding mechanism in ribonucleotides. However, ADA2 is a secreted enzyme, which does not plead for a direct role in intracellular nucleotide production. Mutations in *CECR1* could define a distinct disorder that phenocopies DBA.

## 9. Do Pre-rRNA Processing Defects Contribute to Ribosomopathies?

**Figure 5:**
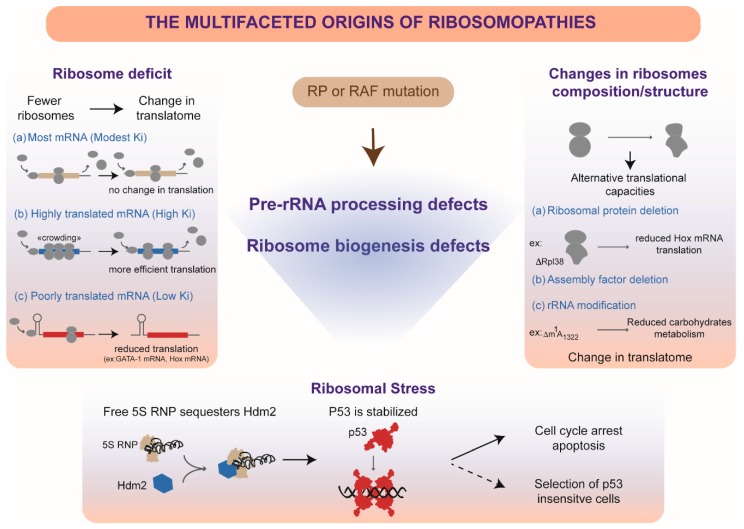
The molecular effects of mutations affecting pre-rRNA processing and ribosome assembly. Mutations in genes encoding RPs or RAFs can lead to pre-rRNA processing or ribosome assembly defects. In human cells, different hypotheses may explain how these defects affect cell fate. Ribosome deficit corresponds to reduction in the number/concentration of ribosomes per cell. Depending on their translation initiation rate (Ki), mRNAs translation may be differently affected: (**a**) Translation of mRNAs that have a modest initiation rate will be mildly affected; (**b**) mRNAs having a high initiation rate may be more efficiently translated, as reduction in ribosome availability will reduce ribosome crowding and thus improve elongation efficiency. (**c**) In contrast, reduced concentrations of ribosomes will penalize translation of mRNAs that have low initiation rates [172]. Ribosome alteration refers to the ribosomes that are still produced despite ribosome assembly defects. Quality of these ribosomes (protein composition, RNA processing, and modification) may be suboptimal and change their translational properties, so that translation of specific mRNAs is not properly ensured or regulated. These different mechanisms are not mutually exclusive and may contribute in several ways to the variety of symptoms presented by patients suffering ribosomopathies. Ribosomal stress is induced by impairment of ribosome biogenesis, which promotes accumulation of free RPs that can assume alternative function. The 5S RNP particle, in which the 5S rRNA associates with RPs RPL5 and RPL11, plays a major role in this stress response. As a free complex, it binds and sequesters HDM2, the ubiquitin ligase that constitutively targets the tumor suppressor p53 for degradation. Inactivation of HDM2 promotes stabilization of p53, which regulates progression through the cell cycle. Chronic stimulation of p53 in ribosomopathies may also favor the selection of cells resisting p53 activity and prone to develop a tumorigenic program.

Other congenital disorders were shown to be linked to mutations in genes encoding ribosome biogenesis factors, including Shwachman–Diamond syndrome (*SBDS*, *DNAJC21*, and *EFL1*), Bowen–Conradi syndrome (*EMG1*), North American Indian childhood cirrhosis (*UTP4*), Treacher–Collins syndrome (*TCOF1*, *POLR1C*, and *POLR1D*), cartilage-hair hypoplasia (*RMRP*), dyskeratosis congenita (*DKC1*, *NOP10*, *NHP2*, and *PARN*), and aplasia cutis congenita (*BMS1*). Depletion of *CIRCH1A*/*UTP4* [154], a SSU processome component, *EMG1* [152,153], *RMRP* [61], *PARN* [39,67], or *BMS1* [156] provokes pre-rRNA processing defects in cultured cells, but whether patients cells display a similar defect was only formally shown so far for *BMS1* mutation [156] (Figure 4 and Table 1). The genes mutated in Treacher–Collins syndrome are involved in rDNA transcription rather than pre-rRNA processing. Similarly, mutations identified in Shwachman–Diamond syndrome affect the last steps of pre-60S particle maturation in the cytoplasm, downstream of all RNA processing events. Finally, proteins synthesized from *DKC1*, *NOP10*, and *NHP2* are components of the snoRNPs, some of which are required for pre-rRNA processing (see above), but there again, the status of pre-rRNA processing or modification in these patients was not directly assessed. While ribosome biogenesis is affected in one way or another by mutations in any of these genes, the contribution of pre-rRNA processing defects per se to each of these diseases is therefore difficult to evaluate. However, a deficit of rDNA transcription upstream of any processing steps, as observed in Treacher–Collins syndrome, leads to a phenotypic spectrum that only partially overlaps with DBA (skeletal abnormalities) and shows no hematopoietic deficit. Pre-rRNA processing defects may thus contribute more specifically to some phenotypes in a subset of ribosomal diseases. Affecting ribosome synthesis can lead to several major and not mutually exclusive molecular consequences (Figure 5). First, mutations in genes encoding RPs or RAFs will reduce the number and/or the quality of the ribosomes, which may impact translation regulation. Second, alterations of ribosome biogenesis can trigger a cellular stress, called ribosomal or nucleolar stress, which affects cell cycle progression independently of translation. In the last parts of this review, we will present the different data that support these hypotheses and comment their relevance to ribosomopathies.

## 10. Insufficient Production of Ribosomes

Pre-rRNA processing defects are very likely to affect the efficiency of ribosome synthesis. Cells tightly regulate their level of ribosomes by controlling their production, a phenomenon called ribosome homeostasis. The amount of ribosomes can vary among different cell types and even different cell stages. For example, a recent study has demonstrated that variations in ribosome homeostasis during circadian rhythm correlate with changes in liver size [170]. In *Xenopus*, ribosome synthesis varies during development and among tissues [171]. Such heterogeneity in ribosome concentration among cell types may influence the repertoire of translated genes depending on the mechanisms regulating their translation, e.g., the translation initiation mode (cap or IRES dependent), the presence of upstream open reading frames (uORFs), the structure of the 5′ untranslated region, or the abundance of rare codons [172]. Hence, it has been proposed that mRNAs with a high initiation rate can be translated at low ribosome concentration, contrary to less efficiently translated mRNA such as IRES containing genes, which would require a higher concentration of ribosomes [172,173]. Therefore, defects in ribosome homeostasis may affect synthesis of specific differentiation or proliferation regulators, which could explain the tissue specificity of phenotypes observed in ribosomopathies (Figure 5). This model is supported by the genetic link established by mutations in DBA between ribosome deficit and defective GATA1 translation [115,118,119]. While DBA appears to be caused in a few patients by mutations in the GATA1 initiation start codon, GATA1 expression is affected in DBA patient cells that bear RP mutation and overexpression of GATA1 in these cells rescues erythroid differentiation [118]. Ribosome profiling experiments have shown that translation of GATA1 mRNA was one of the most sensitive to a drop in ribosome production induced by partial knockdown of different DBA-linked RPs in human CD34^+^ cells [118,119]. Arrest of erythroid differentiation would thus be primarily explained by the negative impact of defective ribosome homeostasis on GATA1 synthesis, irrespective of the haploinsufficient RP. This elegant model provides with a simple explanation as to why the large number of RP genes mutated in DBA are associated with similar clinical outcomes. However, it remains to understand why other disorders potentially affecting the levels of ribosomes, like the Shwachman–Diamond or Treacher–Collins syndromes, do not result in red cell aplasia. Ribosome profiling analysis of tissues from patient suffering from ribosomopathies should help to answer this question.

## 11. Alteration of Ribosome Quality by Pre-rRNA Processing Defects

Recent progress in powerful structural biology technologies such as cryo-EM, mass spectrometry, or RNA analysis by next-generation sequencing (NGS) techniques has provided evidence that ribosomes can be heterogeneous in their composition and/or modifications [48,174,175,176,177]. This heterogeneity comes in support of the idea that subsets of ‘specialized ribosomes’ would be required for the spatiotemporal control of the translation of specific mRNAs, for example during development [46,178,179]. A corollary of this concept is that changes in ribosome quality (rRNA state and/or protein composition) consecutive to defects in ribosome biogenesis may affect the fine-tuned mechanisms of translation and thereby impact tissue-specific biological pathways (Figure 5). Quality of ribosome biogenesis is tightly controlled [180,181], which leads to the degradation of misassembled intermediates [182]. It was shown in yeast that the ultimate steps of the 40S subunit maturation involved a translation-like control, a kind of “test-drive” that ensures that only translationally-competent ribosomal subunits follow the subsequent maturation step [53,183]. In addition, ribosomes defective in translation are also targeted by quality-control mechanisms [184,185,186,187]. These mechanisms, mostly documented in yeast, are expected to limit the risk that aberrant ribosomal subunits impact translation. Nevertheless, it was shown in yeast that particles lacking an RP or bearing misprocessed pre-rRNAs can escape these controls and join the pool of translating ribosomes. Hence, when accumulated in high amounts in yeast expressing RAF mutants, precursors to the 40S and 60S ribosomal subunits still containing pre-RNAs were found among translating ribosomes [187,188,189,190]. While RPS26 is essential for pre-rRNA processing and 40S subunit formation in yeast and humans [40,108], Ferreti et al. engineered yeast producing Rps26-depleted ribosomes and found that such ribosomes preferentially translated mRNAs encoding stress response proteins, most likely by promoting IRES dependent translation [191]. The authors propose that Rps26 normally promotes classical translation initiation by interacting with Kozak sequence residues in the mRNA upstream of the start codon. Whether ribosomes containing immature rRNAs or displaying abnormal RP composition interfere with translation in patients suffering ribosomopathies remains to be demonstrated, but the potential impact of such changes in ribosome quality on the translational capacities of some cells could account for some tissue-specific phenotypes.

Less prone to be targeted by quality control mechanisms, post-transcriptional modifications in pre-rRNAs or post-translational modifications in RPs could also be altered upon pre-rRNA processing defects. As described above, pre-rRNAs undergo a number of post-transcriptional modifications catalyzed by snoRNPs as well as diverse enzymes. These modifications cluster in functional centers of the ribosomal subunits and some are essential for embryonic development in zebrafish. Recent advances in mapping pre-rRNA methylations by NGS have revealed that a subset of them is only present in a fraction of the ribosomes [47,192,193]. These results suggest that the functional properties of a ribosome may be modulated by its RNA modification pattern. Because nucleotide enzymatic modifications are intimately coordinated with pre-rRNA folding and cleavage, defects in pre-rRNA processing could perturb the modification pattern and thereby alter the behavior of ribosomes in translation initiation (selection of translated mRNAs) or elongation (translation fidelity). Ribosome activity also relies on post-translational modification of RPs [194,195,196]. While the cross-talk between these modifications and pre-rRNA maturation is poorly documented, it is easy to conceive that alterations of ribosome biogenesis may also alter protein modifications. Further work is needed to examine the state of rRNA and RP modifications in ribosomopathies and assess their contribution to pathophysiological processes.

## 12. Ribosomal Stress

As described above, many controls are present during ribosome synthesis to ensure degradation of abnormal intermediates in order to avoid their potentially deleterious accumulation. Among the different components of ribosomal particles, some are more resistant than others to degradation and can participate in alternative cellular processes. The 5S particle was recently brought under the spotlight for its role in the regulation of the tumor-suppressor p53. The 5S rRNA-RPL5-RPL11 particle integrates early into pre-60S particles in the nucleolus. However, defects in ribosome synthesis provoke accumulation of 5S RNPs in the nucleus, where they can interact with the ubiquitin ligase HDM2, thus impeding p53 targeting for degradation [99,102]. This leads to p53 stabilization, which in turn promotes cell cycle arrest or apoptosis (Figure 5). Importantly, many phenotypes observed in animal models for ribosomopathies are rescued by p53 knockdown or a p53-null genetic background [197,198]. Thus, developmental problems in ribosomopathies may be explained in part by p53 activation in response to ribosomal stress. In the long run, chronic stimulation of p53 activity could lead to the selection of cells insensitive to p53, which could contribute to the higher susceptibility to cancer observed in several ribosomopathies. Because they directly affect ribosomal assembly and nucleolar organization, pre-rRNA processing defects are expected to trigger ribosomal stress. Other RPs have also been shown to interact with HDM2, but so far only RPL5 and RPL11 have proved essential for p53 stabilization in response to ribosomal stress [199]. However, p53 activation by the 5S RNP is only one component of the nucleolar stress response. Hence, ribosomal stress responses other than p53 stabilization have been evidenced, such as c-Myc activation or p53 translation regulation, but their relevance to ribosomopathies needs to be explored further [200].

## 13. Concluding Remarks on Cross-Talks between Pre-rRNA Processing and Other Gene Expression Processes

As highlighted in this review, human pre-rRNA maturation is a highly complex process intimately linked to the multiple actions required for ribosome production. In recent years, unexpected pre-rRNA maturation steps have been uncovered in mammals compared to yeast, and most nucleases that catalyze pre-rRNA cleavages have been identified. More broadly, proteomic analyses and large-scale genetic screens have revealed the potential involvement of hundreds of factors in human ribosome biogenesis. In addition, the nucleolus, whose formation by self-assembly is driven by ribosome synthesis, also hosts proteins acting in different nuclear processes. The high rate of ribosomal subunit production, the exceptional number of factors involved in ribosome maturation, and the importance of this process in the regulation of cell fate make it very likely that dysfunction of pre-rRNA processing has far-reaching consequences on other cellular processes, starting with genome expression and maintenance. One simple mechanism allowing cross-talk between two different processes is that they share a common enzyme. Indeed, some nucleases involved in pre-rRNA processing are also required for the synthesis or the turn-over of other RNAs, in particular small noncoding RNAs. For example, the 3′–5′ exonuclease PARN, required for the maturation of the 18S-E pre-rRNA, takes part in the maturation of several noncoding RNAs, including snoRNAs, scaRNAs, miRNAs, and the telomerase RNA TERC (see above). The exosome is also involved in the catabolism of several RNAs, including mRNAs [201]. Defects in pre-rRNA processing could perturb the activity of these enzymes in other processes, for example by modifying their bioavailability (e.g., by retention in the nucleolus with misprocessed pre-ribosomal particles) or by altering their capacity to interact with different partners. This could contribute to pathogenic mechanisms in ribosomopathies, as functional defects of both PARN and the exosome were linked to congenital disorders [162,201]. This hypothesis is not restricted to nucleases, and can also be formulated for RAFs [63], and even RPs, some of which have moonlighting activities. It is most likely that the continuous discovery of new links between ribosome synthesis defects and human pathologies will bring important insight into the molecular mechanisms of human ribosome formation and their connection with other cellular processes.

## Figures and Tables

**Table 1:** Pathologies linked to mutations in genes encoding ribosomal proteins or ribosomal assembly factors (ribosomopathies).

Disease Manifestation/Clinical Features	Frequency	Inheritance	Genes Involved in Ribosome Biogenesis	Proteins	Functional Validation of Impact on Ribosome Biogenesis		% of Patients	References
					Patient Cells	Models *		
**Diamond-Blackfan anemia**								
Aregenerative macrocytic anemia, high eADA levels, short stature, craniofacial and upper limb anomalies, heart or genitourinary malformations, predisposition to MDS, AML, and solid tumors	1:150,000–200,000 births	Autosomal dominant	*RPS7*	RPS7	no	yes	<0.1%	[109,120,121,122]
			*RPS10*	RPS10	yes	yes	3%	[123]
			*RPS15A*	RPS15A	yes	yes	<1%	[124]
			*RPS17*	RPS17	yes	yes	1%	[109,125,126,127,128]
			*RPS19*	RPS19	yes	yes	25%	[41,42,129]
			*RPS24*	RPS24	yes	yes	2.4%	[110,130]
			*RPS26*	RPS26	yes	yes	6.6%	[123]
			*RPS27*	RPS27	no	yes	<0.1%	[120]
			*RPS28*	RPS28	no	yes	<0.1%	[114]
			*RPS29*	RPS29	yes	yes	<0.1%	[131]
			*RPL5*	RPL5	yes	yes	7%	[109]
			*RPL11*	RPL11	yes	yes	5%	[109]
			*RPL15*	RPL15	yes	yes	<0.5%	[132]
			*RPL18*	RPL18	yes	yes	<0.1%	[133]
			*RPL26*	RPL26	yes	yes	<0.1%	[134]
			*RPL27*	RPL27	yes	yes	<0.1%	[120]
			*RPL31*	RPL31	yes	yes	<0.1%	[113]
			*RPL35*	RPL35	yes	yes	<1%	[133]
			*RPL35A*	RPL35A	yes	yes	3%	[135]
	One family	X-linked recessive	*TSR2*	TSR2	no	yes	<0.1%	[114]
**5q-syndrome**								
MDS, severe macrocytic anemia	10–15% of patients with MDS or AML	Sporadic	*RPS14*	RPS14	yes	yes	100%	[136]
**Isolated congenital asplenia**								
Absence of spleen, high susceptibility to infections	1:60,000 births	Autosomal dominant	*RPSA*	RPSA	no	yes	100%	[137]
**Other syndromes caused by RP mutations**								
Intellectual disability, autism, microcephaly, hearing loss	Two patients	Autosomal dominant	*RPS23*	RPS23	yes	yes	n.a.	[138]
Autism, microcephaly, mental retardation, growth retardation, seizures, skeletal malformations	Three families/Nine patients		*RPL10*	RPL10	no	yes	n.a.	[139,140,141]
Autism, microcephaly	Two families/Four patients		*RPL10*	RPL10	no		n.a.	[142,143]
Intellectual disability, epilepsy	One patient		*RPL10*	RPL10	no		n.a.	[144]
**Schwachman-Diamond anemia**								
Neutropenia, exocrine pancreatic dysfunction, metaphyseal dysplasia, osteopenia, mild mental retardation, high predisposition to MDS and AML	1:77,000 births	Autosomal recessive	*SBDS*	SBDS	yes	yes	>95%	[145,146]
			*DNAJC21*	DNAJC21 **	yes	yes	2%	[147,148,149]
			*EFL1*	EFL1	no	yes	<0.5%	[149,150]
**Bowen-Conradi Syndrome**								
Growth retardation, psychomotor delay, microcephaly, micrognatia, joint contractures, rockerbottom feet	1:355 in the Hutterite populations	Autosomal recessive	*EMG1*	EMG1	no	yes	100%	[151,152,153]
**North American Indian chilhood cirrhosis**								
Cirrhosis	1:250 in the Ojibway-Cree First Nations population	Autosomal recessive	*CIRH1A*	hUTP4	no	yes	100%	[154,155]
**Familial Aplasia Cutis Congenita**								
Scalp skin defect	1 patient	Autosomal dominant	*BMS1*	BMS1	yes	yes	n.a.	[156]
**Cartilage-hair hypoplasia**								
Hypoplastic macrocytic anemia, neutropenia, defective T-cell, response, short limb dwarfism, fine, sparse hair, skeletal abnormalities, nail dysplasia, gastrointestinal malabsorption, abnormal dentition, predisposition to non-Hodgkin lymphomas and other cancers	1–2:1000 in the Amish population, 1:23,000 in the Finnish population	Autosomal recessive	*RMRP*	-	yes	yes	100%	[61,157]
**Diskeratosis congenita and Hoyeraal-Hreidarsson syndrome**								
Bone marrow failure, pancytopenia, aplastic anemia, mucocutaneous defects, nail dystrophy, developmental delay, pulmonary fibrosis, reduced telomere length, cancer predisposition, immunodeficiency	1:1,000,000 births	X-linked recessive	*DKC1*	Dyskerin	no	yes	25%	[158,159]
		Autosomal recessive	*NOP10*	NOP10	no	no	<1%	[160]
			*NHP2*	NHP2	no	no	<1%	[161]
			*PARN*	PARN	yes	yes	<1%	[162,163]
**Treacher-Collins syndrome**								
Severe craniofacial defects, mental retardation	1:10,000–50,000 births	Autosomal dominant	*TCOF1*	Treacle	no	yes	78–93%	[164]
		Autosomal dominant or recessive	*POLR1D*	RPA16	no	yes	8% ***	[165,166,167]
		autosomal recessive	*POLR1C*	RPA39	no	yes		[165]
**Other RNA polymerase I-related diseases**								
Acrofacial dysostosis	Three patients	Autosomal dominant	*POLR1A*	RPA194	no	yes	n.a.	[168]
Severe neurodegenerative disease, psychomotor retardation, intellectual disability	Two patients		*POLR1A*	RPA194	no		n.a.	[169]

* Tissue culture of mammalian cells or vertebrate models; ** DnaJ homolog subfamily C member 21; *** Combined % for *POLR1D* and *POLR1C* mutations. eADA, erythrocyte adenosine deaminase; MDS, Myelodysplastic syndrome; AML, Acute myeloid leukemia; n.a., not available.
